# Communication Apprehension and Eye Contact Anxiety in Video Conferences Involving Teleoperated Robot Avatars: A Subjective Evaluation Study

**DOI:** 10.3389/frobt.2021.758177

**Published:** 2021-11-05

**Authors:** Faisal Mehmood, Hamed Mahzoon, Yuichiro Yoshikawa, Hiroshi Ishiguro

**Affiliations:** ^1^ Intelligent Robotics Lab, Department of Systems Innovation, Graduate School of Engineering Science, Osaka University, Osaka, Japan; ^2^ Institute for Open and Transdisciplinary Research Initiatives (OTRI), Osaka University, Osaka, Japan

**Keywords:** communication apprehension, eye contact anxiety, sense of being attended, video teleconference, robotic video teleconference, robot avatar in video conferences

## Abstract

Communication apprehension (CA), defined as anxiety in oral communication, and anxiety in eye contact (AEC), defined as the discomfort felt in communication while being stared at by others, limit communication effectiveness. In this study, we examined whether using a teleoperated robot avatar in a video teleconference provides communication support to people with CA and AEC. We propose a robotic telecommunication system in which a user has two options to produce utterance for own responses in online interaction with interviewer i.e., either by a robot avatar that faces the interviewer, or by self. Two imagination-based experiments were conducted, in which a total of 400 participants were asked to watch videos for interview scenes with or without the proposed system; 200 participants for each experiment. The participants then evaluated their impressions by imagining that they were the interviewee. In the first experiment, a video conference with the proposed system was compared with an ordinary video conference, where the interviewer and interviewee faced each other. In the second experiment, it was compared with an ordinary video conference where the interviewer’s attentional focus was directed away from the interviewee. A significant decrease in the expected CA and AEC of participants with the proposed system was observed in both experiments, whereas a significant increase in the expected sense of being attended (SoBA) was observed in the second experiment. This study contributes to the literature in terms of examining the expected impact of using a teleoperated robot avatar for better video conferences, especially for supporting individuals with CA and AEC.

## Introduction

Communication apprehension (CA) is defined as “an individual’s fear or anxiety associated with either real or anticipated communication with another person or persons” (McCroskey, 1982). This anxiety not only affects the daily life communication of an individual in face-to-face (FtF) interactions (Elwood and Schrader, 1998; Thomas et al., 1994; Blume et al., 2013; Drinkwater and Vreken, 1998) but also their online interactions (Punyanunt-Carter et al., 2018; Ho and McLeod, 2008). CA reduces the communication effectiveness of an individual (Freimuth, 1976) and may lead others to perceive them as a less positive communication partner (McCroskey and Richmond, 1976). People with CA avoid communication through nonverbal behaviors such as fewer kinesic movements, longer normative pauses, and reduced eye contact (McCroskey, 1976). Conversely, anxiety in eye contact (AEC) refers to the feeling of discomfort or fear that a person feels while being stared at by others (Schulze et al., 2013). Social anxiety may generate AEC in an individual (Schneier et al., 2011); AEC reduces eye contact duration and frequency (Moukheiber et al., 2010), which ultimately affects both daily life FtF communications (Hodge, 1971; Argyle and Dean, 1965) and online communications (Howell et al., 2016).

Audio and text-only technologies, such as online social websites, cell phones, text/instant messaging (Pierce, 2009), audio telephonic calls, voice mail, electronic mail (Rice, 1993), and computer-mediated communications (CMC) (Thurlow et al., 2004) are available as alternatives to FtF interactions. Such alternative technologies for communication moderate the social anxiety of users (High and Caplan, 2009) and are preferred by individuals with social anxiety and CA (Pierce, 2009; Reinsch and Lewis, 1984). However, such alternatives have removed the opportunities for eye contact, which has made communication non-vivid. These technologies also reduce the social presence of users (Short et al., 1976; Oh et al., 2018; Borup et al., 2012), defined as the perception of an individual’s presence in the communication (Calefato and Lanubile, 2010). Reduced social presence is one of the causes for the failure to maintain the sense of being attended (SoBA) in the users. SoBA is defined as the feelings experienced by the participant when listened to, given attention, focused upon, or questioned/answered by others in conversations.

Video conferencing is another alternative technology to FtF interactions that reduces the CA and AEC of people, (Leeds and Maurer, 2009; Sautter and Zúñiga, 2018; Scott and Timmerman, 2005) while maintaining social presence (Keil and Johnson, 2002; Ko, 2016). People prefer it over audio-only technology, because it provides the participants information that are both verbal and nonverbal, such as details about the remote partners’ attentional focus (Daly-Jones et al., 1998). This would contribute to establishing mutual understanding (Isaacs and Tang, 1994). However, video conferences may lead to unnecessary eye contact opportunities that produce anxiety (Bohannon et al., 2013), fear-relevant features (Wieser et al., 2009), gaze avoidance behaviors (Weeks et al., 2013), and interrupted dialogs (O’Malley et al., 1996). To avoid the AEC problem for users with CA in video technology, an interlocutor can be instructed to avert gaze during interaction. However, averting gaze alone is not effective in regulating the participant’s anxiety (Langer and Rodebaugh, 2013). Moreover, this stratagem reduces their social presence in video conferences (Bondareva et al., 2006).

Avatars are “an interactive, social representation of a user” (Meadows, 2007) or a representation of oneself in a given physical medium for experiencing the physical environment (Castronova, 2003). Avatars can be either virtual or physical ones; where virtual avatars are graphical or digital representation of users in virtual environments, while physical avatars are embodied representations of users in real environments (Aljaroodi et al., 2019). Robot avatars have been found to be effective for various online communication situations, including education (Børsting and Culén, 2016), (Shimaya et al., 2019), virtual tours of different locations (Cheung et al., 2017), and family communication (Lee et al., 2009). Previous studies have shown that using robot avatars masks the identity of the user (Straub et al., 2010; Choi and Kwak, 2017), which would contribute to reducing CA and AEC. Meanwhile, it was shown that the user could enhance own social presence with a physical robot avatar (Tanaka et al., 2015; Gleason and Greenhow, 2017). Considering the advantages of using physical robot avatars for interactions, we expect that placing a physical robot beside the interlocutor in the video conference as the user’s avatar reduces the user’s AEC while maintaining SoBA. In such a scenario, the attentional focus of the interlocutor is shifted to the robot avatar, which contributes to reducing the user’s AEC. Further, it is expected that the user will not lose SoBA by the interlocutor because the focus of the interlocutor’s attention is directed to user’s own avatar.

**FIGURE 1:**
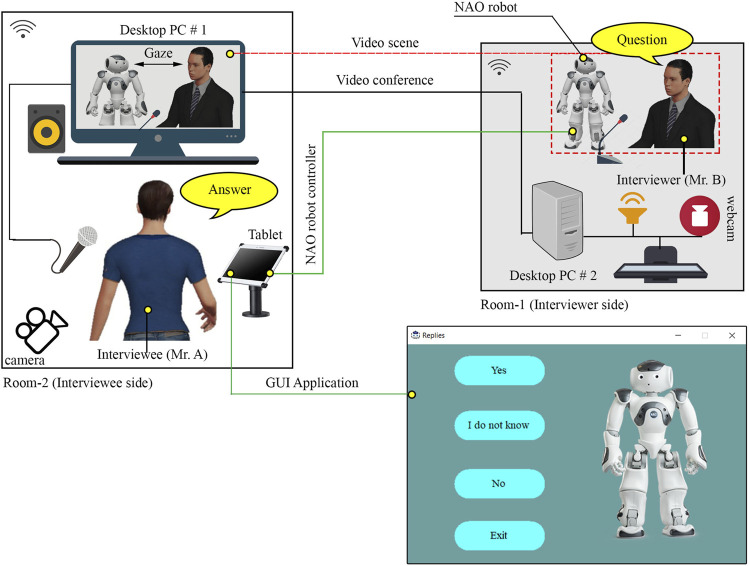
Robot condition (with interviewer’s gaze toward the robot avatar).

Therefore, in this study, we propose a robotic system to support a user with CA and AEC in a conversation in tele-communication. Assume a situation where the user is involved in an online discussion with an interlocutor through a humanoid robot teleoperated by the user and placed at the interlocutor’s side (see Figure 1). The user can see the interlocutor and the profile of teleoperated robot avatar on the monitor in real time. Hereafter, we denote the physical avatar implemented as a physical robot be the robot avatar. Such a system enables two options for the user: utterance through the robot avatar and utterance by self. Consequently, the interlocutor also has two options: directing attention to the robot avatar of the user and the user’s image on the monitor. These situations are expected to reduce the user’s CA and AEC by decreasing the attentional focus by the interlocutor on the user. At the same time, it is expected that the user can maintain SoBA even if the interlocutor’s attention is often directed to the robot because it is expected to feel realistic; felt like an avatar of own self. To verify such effects, the participants watched videos including scenes of telecommunication with or without the proposed system, after which two different video evaluation experiments were conducted: They were asked to imagine that they were the user in the videos; their expected CA, AEC, and SoBA were evaluated. In Experiment-Ⅰ, the video for the proposed method was compared with one that included a scene of an ordinary online conversation system where the user observed the frontal face of the interlocutor to evaluate the effects of the proposed method on their expected CA and AEC. In Experiment-Ⅱ, it was compared with another, including a scene with an online conversation system where the user often observed the profile face of the interlocutor to evaluate the effects of the proposed method on their expected SoBA.

## Robotic Video Teleconferencing System for Providing Communication Support to People With CA and AEC

Figure 1 shows a schematic of the proposed system. It consists of a desktop computer, tablet, and humanoid robot. Using the desktop computer, an online discussion session was arranged between a human interviewer and an interviewee physically present at different locations (Room-1 and Room-2, respectively). The robot beside the interviewer in Room-1 was controlled by the interviewee in Room-2 with the tablet. The interviewee could observe both the robot and the interviewer in Room-1 with a commercial software for the online video conferences. We used the NAO robot: a bipedal robot with 25 degrees of freedom; height of 58 cm; programmable in multiple languages; and capable of producing visual, speech, and motion stimuli for interaction. Throughout the interviews, the robot was in a standing position with subtle idling movements: gentle left and right movements without changing the position of its feet on the table. It alternately looks at the interviewer and interviewee by turning its head. It looks at the camera on the screen in the online conference in Room-1 to be perceived as looking at the interviewee in Room-2 due to the Mona Lisa effect; an illusion effect where a person in the image is perceived by others as gazing at them, regardless of their position relative to image (Horstmann and Loth, 2019). The GUI on the tablet consists of four buttons: “yes,” “no,” “I do not know,” and “exit.” The server–client architecture of the transmission control protocol (TCP) was used to exchange the information (commands) between the tablet and robot over the local network. The TCP client role was integrated in the robot and GUI of the tablet, whereas for the TCP server role, a separate executable file runs on desktop pc # 2. As soon as the interviewee pushes a button on the tablet, the robot stops the idling motion, turns its head toward the interviewee, nods twice, turns back to the interviewer, and utters any of the following: “yes, I think I will,” “no, I think I do not,” and “I do not know,” corresponding to the buttons “yes,” “no,” and “I do not know,” respectively. Note that the “exit” button is used to terminate the operation of robot but has not been used in this study. In the conversation using this system, the interviewer asks the robot a yes/no question followed by an in-depth question. The interviewee was assumed to answer the yes/no question through the robot using the tablet and the in-depth question using own voice. Answering the yes/no questions is expected to be easier for the interviewee than concisely explaining about thoughts.

## Experiment-I

### Materials and Method

#### Method

**FIGURE 2:**
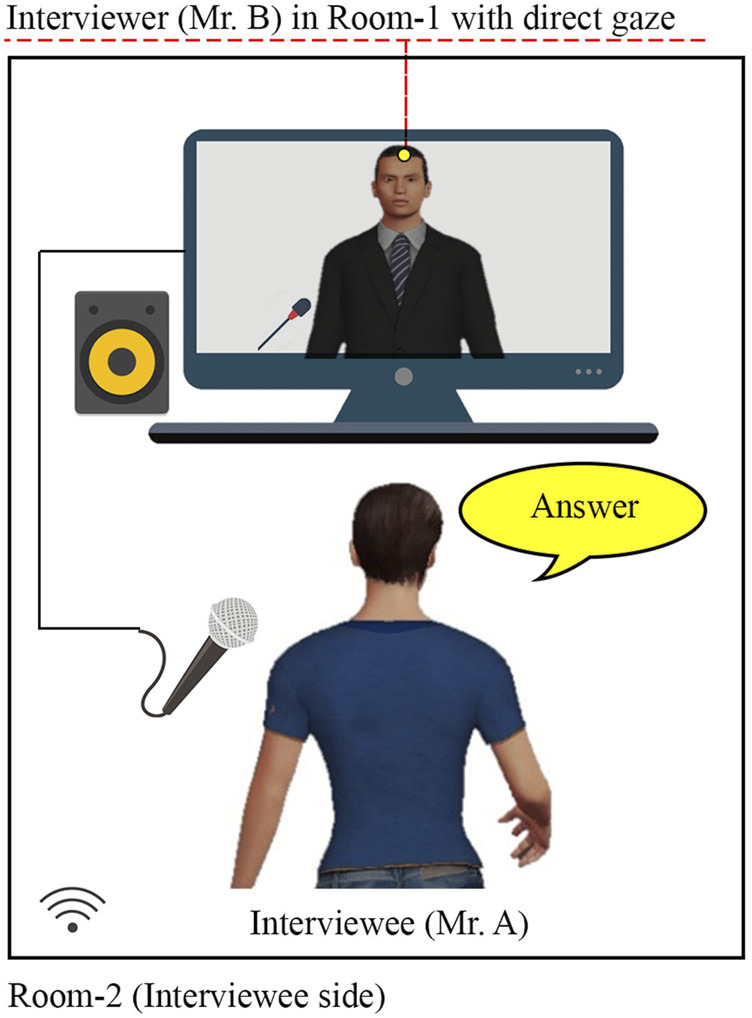
Human condition of Experiment-Ⅰ (with interviewer’s gaze toward the interviewee).

The interviewee’s perspective of the conversation using the proposed system [Robot condition (see Figure 1)] was compared to their perspective of the one without the system [Human condition (see Figure 2)]. This study involved a web-based survey system: instead of a direct interviewee’s experience, the participants were asked to watch the video clips of conversations. The conversations included both Human and Robot conditions (independent variables). The participants later evaluated their perceived CA, AEC, SoBA, and intention to use (ITU) (dependent variables) by imagining themself to be the interviewee.

#### Participants

We recruited 200 participants [Mean age (M) = 32.73 years, SD = 8.96] through the Internet. The participants included 158 males and 42 females, with no serious CA and AEC; they were divided into two groups, G1 and G2, based on their date of birth (even = 113, odd = 87).

#### Apparatus

The participants used a web browser interface to watch the recorded video conversations for both conditions and answered the questionnaire described in *Survey* Section.

#### Stimuli

**FIGURE 3:**
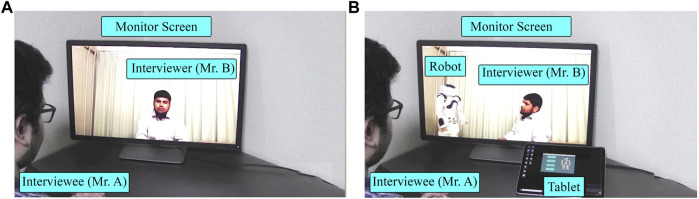
Pictures from video stimuli of Experiment-Ⅰ: **(A)** Human condition (with interviewer’s gaze toward the interviewee.); **(B)** Robot condition (with interviewer’s gaze toward the robot avatar, controlled by the interviewee).

Conversations (in both conditions) between two experimenters were related to topics of earning money through unfair means and paying taxes. In the Human condition, an ordinary video conference system namely Zoom, (Zoom Video Communications Inc. 2011) was used, where the interviewer’s gaze was directed at the monitor with a web camera so that the interviewee in Room-2 would perceive the interview as directed by the interviewer (see Figure 3A). In the Robot condition, the interviewer’s gaze was directed at the robot throughout the conversation except when interviewer shortly glanced at the interviewee to invite answers to in-depth questions (see Figure 3B). The video stimuli lasted 38 and 51 s for the Human and Robot conditions, respectively. The latter was longer than the former because of the robot’s delay to utter yes/no answers. In both video stimuli, the sequence of utterances remained identical. The interviewer asked two questions: a yes/no question followed by an in-depth question. The interviewer’s questions and interviewee’s answers in the video stimuli are given in Supplementary Appendix S2.

#### Manipulation Check

Two manipulation checks were performed to verify whether the participants carefully watched and understood the content of the video stimuli for each condition. Further analysis was performed on the participants’ data to verify if they passed both the manipulation checks.

#### Survey

The participants completed an online survey form consisting of six parts. In part Ⅰ, participants were required to read and agree with the content of web-based informed consent. Some personal details such as age, gender, and daily life CA and AEC were obtained in Parts Ⅱ and Ⅲ. Information about daily life CA (M = 16.85, SD = 4.57) and AEC (M = 44.18, SD = 25.15) was obtained to check serious issues, if any, in the participants. The G1 participants watched a Human condition interview in part Ⅳ (Figure 3A) and Robot condition interview (Figure 3B) in part Ⅴ. Immediately after watching each of them, they were asked to imagine and rate their perceived CA, AEC, and SoBA. In G2, the order was reversed. Finally, participants were asked about their preference of the Human and Robot conditions when the interlocutor was their boss, teacher, doctor, psychologist, or stranger.

#### Measurements

##### Expected Communication Apprehension

The participants’ response to CA was recorded three times in a web-based survey, namely in Parts Ⅲ, Ⅳ, and Ⅴ, using the interpersonal sub-score of personal report of communication apprehension-24 (PRCA-24) (McCroskey, 2015). A 1–5 Likert-type point scale was used (strongly disagree, disagree, neither agree nor disagree, agree, and strongly agree).

##### Expected Anxiety in Making/Avoiding Eye Contact

The participants’ responses to the AEC questionnaire were recorded in Parts Ⅲ, Ⅳ, and Ⅴ of web-based survey using the gaze anxiety rating scale (GARS) (Schneier et al., 2011). A 0–3 Likert-type point scale was used (none, mild, moderate, severe), where ratings are summed to yield the total score.

##### Expected Sense of Being Attended

We developed a scale named SoBA that quantifies the feelings of an individual when being listened to, given attention, focused upon, or questioned/answered by others in conversations; see Supplementary Appendix S1. The participants were asked to imagine and rate how much SoBA they expected to have if they were the interviewee in the video. It was obtained two times in the web-based survey (Parts Ⅳ and Ⅴ), with the 1–5 Likert-type point scale. This index is made to fit with this experiment; therefore, its internal consistency is reported in the Results section.

##### Intention to Use the System

To evaluate an individual’s intention to use the video conferencing system in the Robot condition, the intention to use (ITU) questionnaire (Heerink et al., 2010) with a 1–5 point scale was used at the end of the web survey (part Ⅵ).

##### Preference to Use the System

The preference of an individual to use the video conferencing system was evaluated in the Robot condition, where the interlocutor is individual’s own boss, teacher, doctor, psychologist/counselor, or a stranger. It was also evaluated by simply asking their degree of agreement in using it in each situation on a 1–5 point scale.

### Results

#### Expected Communication Apprehension

**FIGURE 4:**
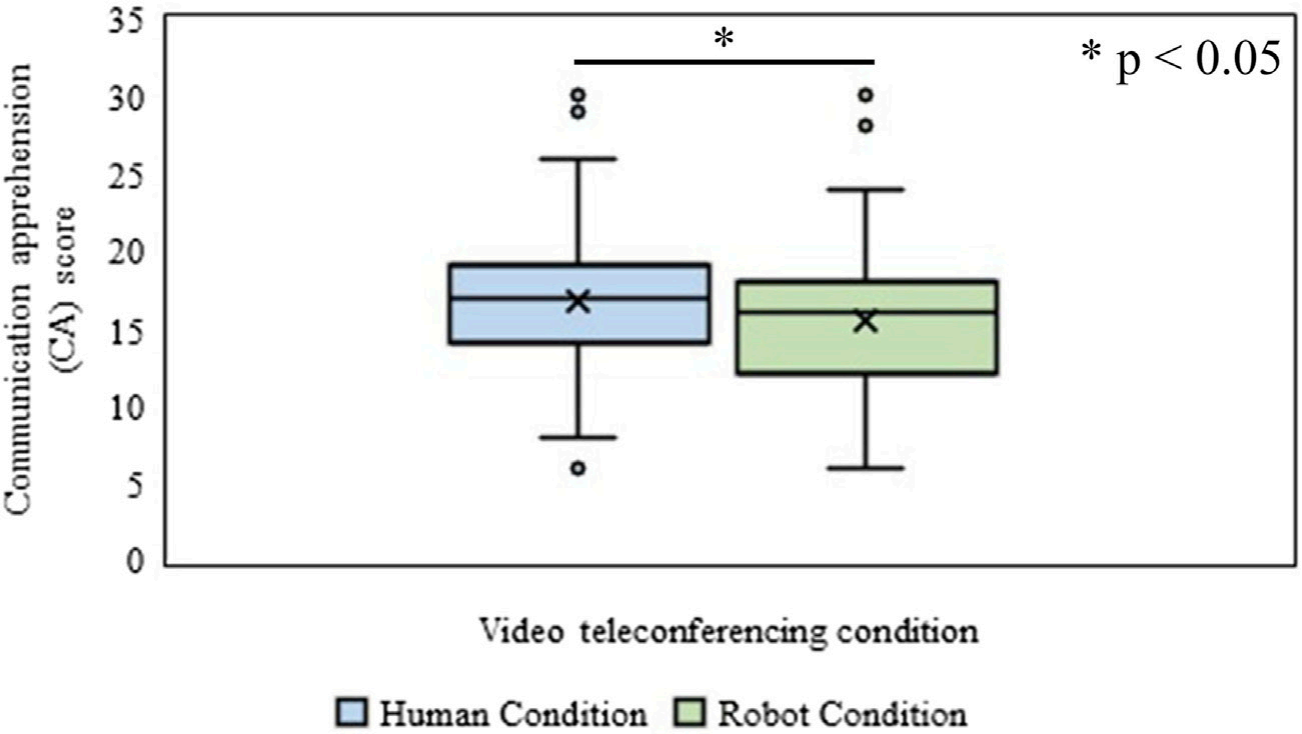
Communication apprehension (CA) in Human and Robot conditions of Experiment-Ⅰ.

The Wilcoxon signed-rank (WSR) test was conducted to identify the effect of the type of video conferences (Human vs. Robot conditions) on the expected CA of the participant. It was revealed that the mean rank of expected CA of the participant for the human condition was significantly higher (*Mdn* = 17) than that in the Robot condition (*Mdn* = 16), (*n* = 200, Z = 3.71, *p* = 2.08 × 10^−4^, *r* = 0.18), (Figure 4). The *p*-values reported in this paper are two-tailed.

#### Expected Anxiety in Making/Avoiding Eye Contact

**FIGURE 5:**
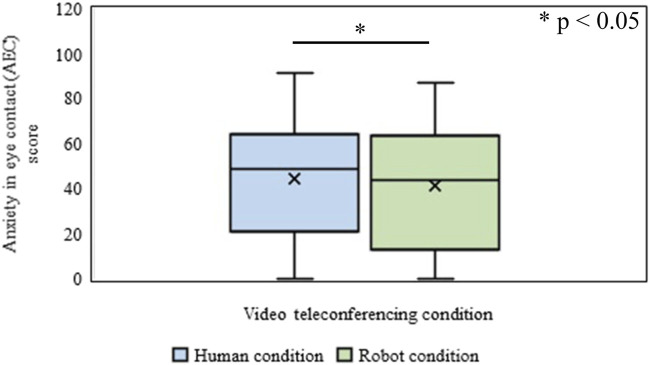
Anxiety in eye contact (AEC) in Human and Robot conditions of Experiment-Ⅰ.

The effect of the type of video conference (Human vs. Robot conditions) on the excepted AEC of the participant was identified through the WSR test. It was revealed that the mean rank of the expected AEC of the participant for the human condition was significantly higher (*Mdn* = 49) than that in the Robot condition (*Mdn* = 44) (*n* = 200, Z = 3.37, *p* = 7.27 × 10^−4^, *r* = 0.17) (Figure 5).

#### Expected Sense of Being Attended

**FIGURE 6:**
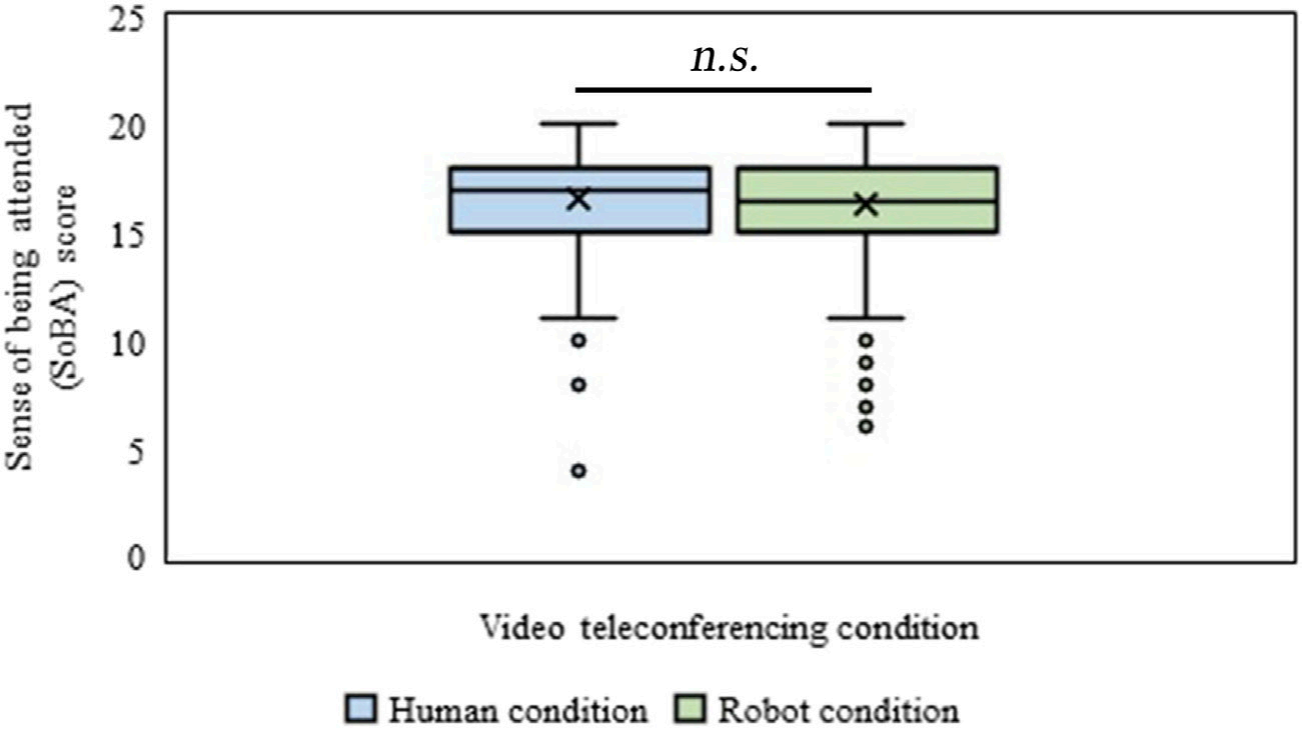
Sense of being attended (SoBA) in Human and Robot conditions of Experiment-Ⅰ.

The effect of the type of video conferences (Human vs. Robot conditions) on the expected SoBA of the participant was identified through the WSR test. There was no significant difference between the mean rank values of the expected SoBA of the participant for Human (*Mdn* = 17) and Robot (*Mdn* = 16.5) conditions (*n* = 200, *Z* = 0.44, *p* = 0.65, *r* = 0.022), (Figure 6). The internal consistency of the SoBA scale, which we used in this experiment, was high (*α* = 0.81).

#### Intention to Use the System

The WSR test for a single sample, using *hypothesized Mdn* = 3.0, which was the center value for this scale, showed a significantly higher tendency of the participants to use the Robot condition (*Mdn* = 4.0); *n* = 200, *Z* = 6.51, *p* = 7.36 × 10^−11^, *r* = 0.46.

#### Preference to Use the system

Friedman’s test identifying the effect of the type of role of the interviewer on the preference of the interviewee for using the Robot condition revealed no significant effect: *χ* ^*2*^(4, *n* = 200) = 9.44, *p* = 0.051.

## Experiment-II

### Materials and Method

#### Method

**FIGURE 7:**
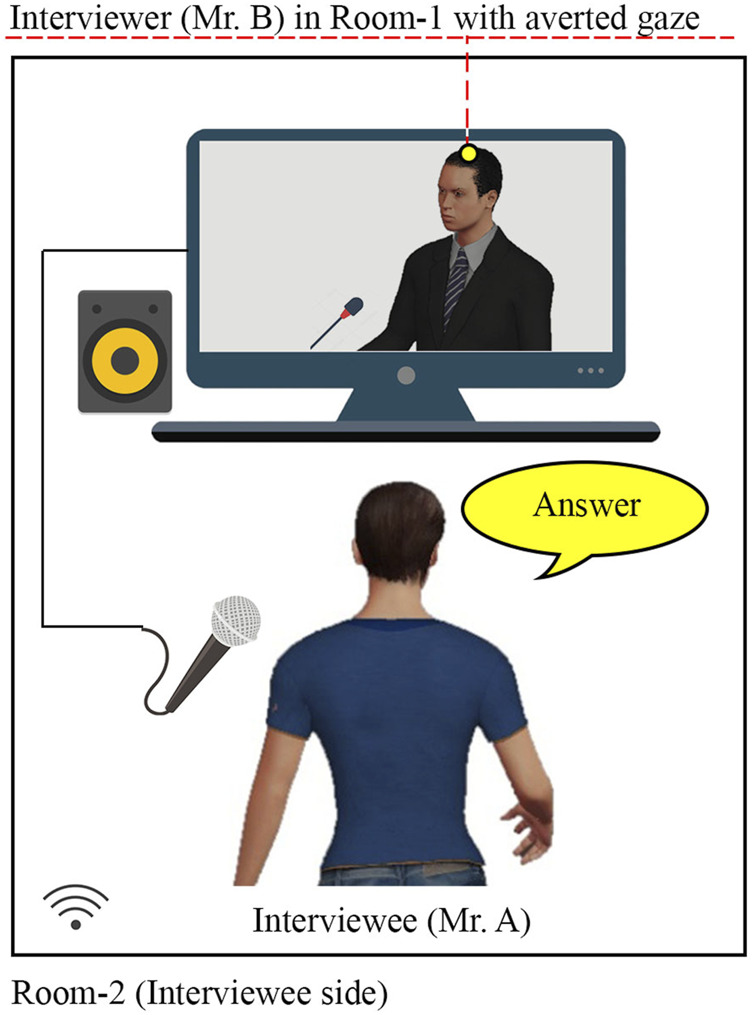
Human condition of Experiment-Ⅱ, (with interviewer’s gaze averted from the interviewee).

**FIGURE 8:**
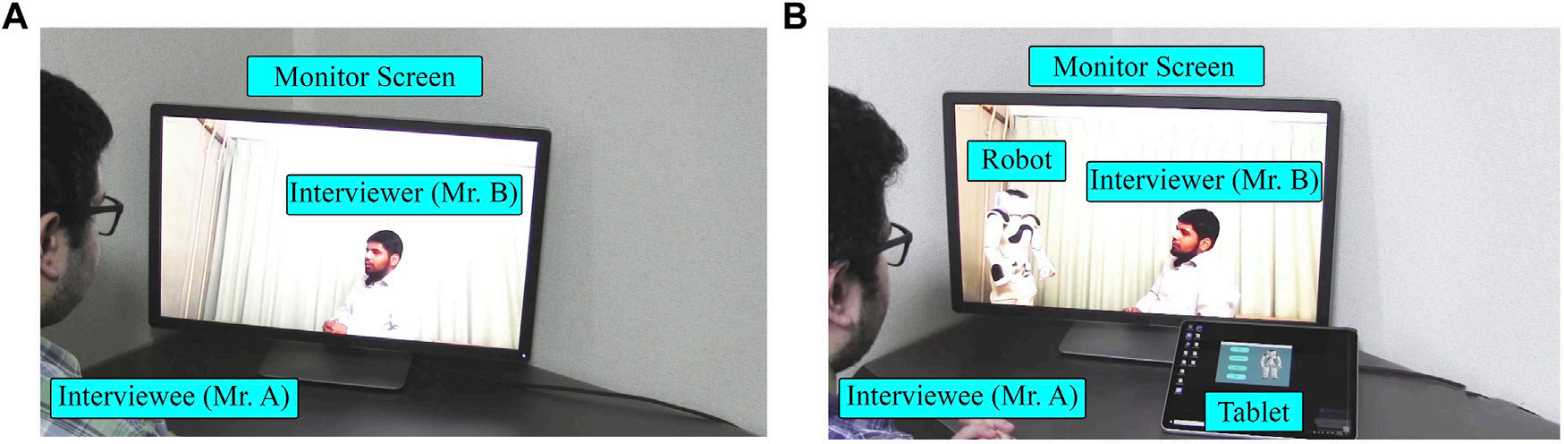
Video stimuli from Experiment-Ⅱ: **(A)** Human condition (with averted interviewer gaze); **(B)** Robot condition (with interviewer’s gaze toward robot avatar controlled by the interviewee).

The results of Experiment-Ⅰ showed a positive effect of the proposed system on the participants’ CA and AEC. The interviewer’s gaze was not controlled in Experiment 1; therefore, the positive effect was possibly caused simply by the interviewer’s gaze pattern averting from the interviewee in the Robot condition. If this is true, such a simple behavioral strategy of the interviewer may be sufficient for reducing the interviewees’ CA and AEC. However, in addition to reduction in CA and AEC, the averted gaze of the interviewer may also reduce SoBA. Experiment-Ⅱ was conducted to further verify the difference between the Robot and Human condition with a new interviewer who showed different patterns of gaze. In the new condition, called Human (averted) condition, the interviewer’s gaze was directed away from the monitor with a web camera so that the interviewee perceived the interviewer as looking away (see Figures 7, 8A). Note that the interviewer’s gaze in Human (averted) condition was controlled such that the relative angle of the gaze to the center was the same as that in the Robot condition (Figure 8B).

#### Participants

A different set of 200 participants [Mean age (*M*) *=* 32.66 years*, SD* = 9.29] was recruited from the Internet. The participants included 148 males and 52 females, with no serious CA (M = 17, SD = 3.63) and AEC (M = 49.88, SD = 24.23); they were divided into two groups, G1 and G2, based on their date of birth (even = 128, odd = 72).

#### Apparatus

The participants used a web browser interface to watch the video stimuli in both conditions and answered the questionnaire.

#### Stimuli

In the Human (averted) condition, the same conversation content used in Experiment-Ⅰ was adopted (see Supplementary Appendix S2), except for the gaze pattern of the interviewer (see Figure 8A). The duration of the video stimuli were 39 and 51 s for the Human (averted) and Robot conditions, respectively.

#### Manipulation Check

The same manipulation checks, which were used in Experiment-Ⅰ, were used, and the participants who passed them were considered for data collection.

#### Survey

The procedure for Experiment-Ⅱ was identical to that of Experiment-Ⅰ, except for the video stimulus used in the Human condition.

#### Measurements

The measurements used were the same as that of Experiment-Ⅰ.

### Results

#### Expected Communication Apprehension

**FIGURE 9:**
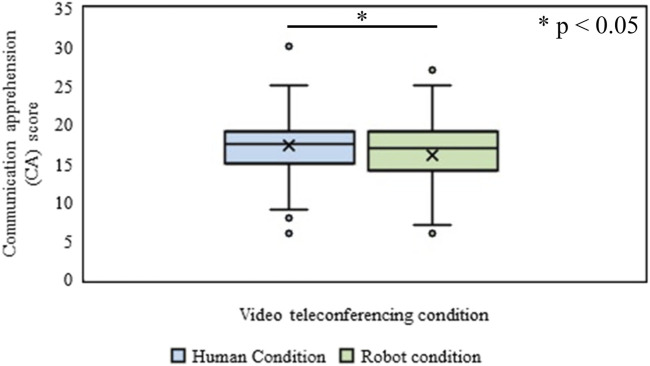
Communication apprehension (CA) in Human and Robot conditions of Experiment-Ⅱ.

The effect of the type of video conferences (Human vs. Robot conditions) on the expected CA of the participant was identified through the WSR test. It showed that the mean rank of the expected CA of the participant for the Human condition was significantly higher (*Mdn* = 17.5) than that in the Robot condition (*Mdn* = 17), (*n* = 200, *Z* = 3.38, *p* = 7.2 × 10^−4^, *r* = 0.17), (Figure 9).

#### Expected Anxiety in Eye Contact

**FIGURE 10:**
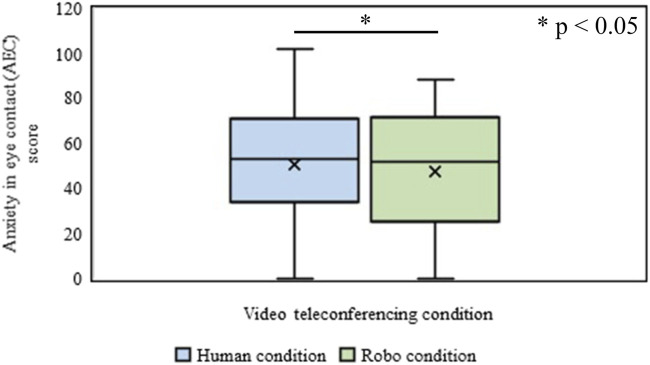
Anxiety in eye contact (AEC) in Human and Robot conditions of Experiment-Ⅱ.

The effect of the type of video conferences (Human vs. Robot conditions) on the expected AEC of the participant was identified through the WSR test. It showed that the mean rank of the expected AEC of the participant for the Human condition was significantly higher (*Mdn* = 53) than that in the robot condition (*Mdn* = 52) (*n* = 200, *Z* = 2.04, *p* = 0.040, *r* = 0.10), (Figure 10).

#### Expected Sense of Being Attended

**FIGURE 11:**
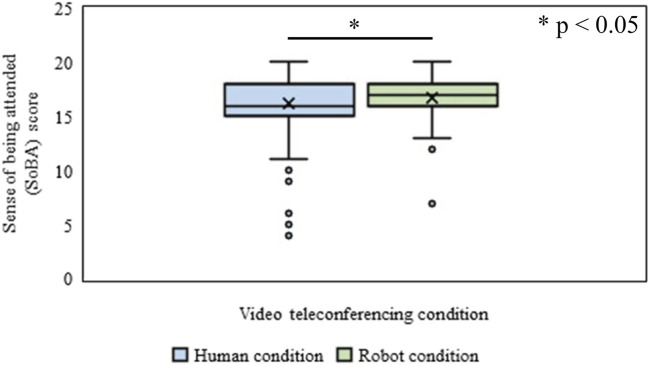
Sense of being attended (SoBA) in Human and Robot conditions of Experiment-Ⅱ.

The effect of the type of video conferences (Human vs. Robot condition) on the expected SoBA of the participant was identified through the WSR test. It showed that the mean rank of the expected SoBA of the participant for the Human condition was significantly lower (*Mdn* = 16) than that in the Robot condition (*Mdn* = 17), (*n* = 200, *Z* = 2.39, *p* = 0.016, *r* = 0.12), (Figure 11).

#### Intention to Use the System

The WSR test for a single sample, using *hypothesized Mdn* = 3.0, which was the center value for this scale, showed a significantly higher tendency of the participants to use the Robot condition (*Mdn* = 4.0); *n* = 200, *Z* = 8.18, *p* = 5.81 × 10^−18^, *r* = 0.58.

#### Preference to Use the System

Friedman’s test for identifying the effect of the types of roles of interviewer on the preference of interviewee for using Robot condition showed a significant effect; *χ* ^*2*^(4, *n* = 200) = 16.30, *p* = 0.003. Multiple WSR tests with Bonferroni corrections revealed a significant preference for using the Robot condition for communicating with bosses (*Mdn* = 4.0, *SE* = 0.070) over doctors (*Mdn* = 4.0, *SE* = 0.073) (*n* = 200, *Z* = −2.249, *p* = 0.025, *r* = −0.11); with teachers (*Mdn* = 4.0, *SE* = 0.081) over doctors (*Mdn* = 4.0, *SE* = 0.073), (*n* = 200, *Z* = −2.708, *p* = 0.007, *r* = −0.14); and teachers (*Mdn* = 4.0, *SE* = 0.081) over strangers (*Mdn* = 4.0, *SE* = 0.074), (*n* = 200, *Z* = −2.220, *p* = 0.026, *r* = −0.11).

## Discussion

In Experiment-Ⅰ, a significant reduction in the expected CA and AEC was observed in the Robot condition, showing that the robot avatar utilization as a medium of communication in a video conference provides communication support by regulating the participants’ CA and AEC. However, it was not clear whether the shifted attentional focus of the interviewer from the interviewee is sufficient to support individuals with CA and AEC. In Experiment-Ⅱ, the reduction of the expected CA and AEC in the Robot condition compared to the Human (averted) condition implies that using a robot can enhance the effect by merely shifting the attentional focus of the interviewer. Furthermore, the averted gaze of the interlocutor reduced the SoBA in the Human (averted) condition, however not in the Robot condition. In other words, a mere shift in the attentional focus of the interviewer interferes with the interviewees’ social presence in video conferences. Therefore, it is suggested that the use of a teleoperated robot avatar as a medium of communication in video conferences provides communication support: it helps in reducing the anxieties of the user while maintaining the SoBA.

Perception of direct gaze of interlocutors generates fear-relevant features in people with social anxiety (Wieser et al., 2009). The reduced CA and AEC in the Robot condition of Experiment-Ⅱ could be explained by the fact that there was a chance to explicitly perceive that the attentional focus of the interviewer was directed at a different agent (the robot in the proposed system). This contributes to reducing such fear-relevant features. Conversely, in online interaction, shared gaze toward a specified area in a scene is known to increase engagement among the participants (Maurer et al., 2018). There was a consistent chance for the interviewee to share attention with the interviewer through the robot in the proposed system. Furthermore, considering that the event observed through the avatar is perceived as being as the operator’s own experience (Morita et al., 2007), the perception of engagement is also possibly enhanced. This is reinforced by perceiving the eye contact between the interviewer and the avatar as occurring between the interviewer and interviewee, without apprehension. Moreover, as the perception of the averted gaze of interlocutors activates interaction avoidance behaviors (Hietanen et al., 2008), the enhanced experience of eye-contact or non-averted gaze would contribute not only in enhancing the SoBA, but also in motivating them to actively communicate with the interlocutor. These potential merits for the interviewee are considered to result in a high ITU for the proposed system.

Despite the communication support through the teleoperated robot avatar in video conferences, there are limitations. First, having significant statistical differences does not necessarily mean a large change/improvement. In addition, the found effects were observed in the pre-recorded videos, which might not be necessarily guaranteed to be reproduced in real-world use. Therefore, further interactive studies are required. The results were imagination-based evaluation, where the recruited participants were asked to imagine themselves as an interviewee in the video scenes they watched. However, the degree to which a participant could imagine own self as the character in a scene was not controlled. Moreover, we did not recruit participants who had severe CA and AEC and were eager to be supported in a video conference. Therefore, it is essential to evaluate the effectiveness of the proposed method as a treatment for individuals with such anxieties, especially in real world interview scenarios. To overcome the current limitation of the results only with mere significant difference in the imagination-based experiment with individuals without severe CA and AEC, interactive experiments with individuals affected with severe CA and AEC using the proposed system to communicate with others are required to observe the actual potential of the system at a practical level and to draw more affirmative conclusions. In addition, to be used in the real-world support, it is considered that the proposed system is expected to be interested and acceptable not only for the interviewee but also for interviewer who might suffer from the difficulties of the interviewee’s CA and AEC. Although, for the simplicity, we coped with the expected effect in the interviewee’s side, it should be worth examining what effects are expected in the interviewer’s side. Although we supposed that giving the limited number of pre-defined answer to candidate like yes/no is a supportive way for individuals with CA and AEC to respond to questions, however, it simultaneously limits the freedom of conversation. To relax such limitation, it is worth studying the user-friendly or automatic mechanism to dynamically change the answer candidates based on the technologies such as one to predict next probable words for the input sentences like chatterbots (Chakrabarti and Luger, 2015; Ashktorab et al., 2019). Other than limitations, some challenges might be experienced during the integration of the proposed system in the real world. In the beginning, it might be a challenging task to find the appropriate individuals having both severe CA and AEC and later to train them for the usage of the proposed system in daily life. Further, in subsequent stages, it might also be challenging to endure the cost of deployment of the system and later bear the maintenance cost along with multiple unforeseen technical issues for which individuals with CA and AEC will be completely dependent on service providers.

## Conclusion

In this research, we proposed and demonstrated that using a teleoperated robot avatar in a video conference provides communication support to people experiencing CA and AEC. The evaluations were imagination-based, where the participants were asked to watch videos of interview scenes with or without the proposed system and evaluate their impressions by imagining they were the interviewee. In the proposed system, the interviewee had two options: utterance by a robot avatar that faced the interviewer, and utterance by self. Practically, a video conference with a teleoperated robot avatar was compared with an ordinary interview (interviewer’s gaze directed at the interviewee) and another, where the attentional focus of the interviewer was diverted from the interviewee. Experimental results showed the positive effect of the proposed method on the expected CA, AEC, and the social presence of the interviewee. This study contributes to the literature in terms of examining the expected impact of using robot avatars in video conferences to provide communication support to people with CA and AEC. It also contributes toward establishing better video conferences. In the future, to overcome the limitations of the imagination-based experiment with individuals without severe CA and AEC, we will examine whether using a teleoperated robot avatar provides communication support in interactive experiments, including potential users with severe CA and AEC, and with different cultural and linguistic backgrounds.

## Data Availability

The original contributions presented in the study are included in the article/Supplementary Material, further inquiries can be directed to the corresponding author.
