# Supplementary material for: Communication Apprehension and Eye Contact Anxiety in Video Conferences Involving Teleoperated Robot Avatars: A Subjective Evaluation Study
Source: Front Robot AI. 2021 Nov 5;8:758177. doi: 10.3389/frobt.2021.758177 (PMC8602855; doi:10.3389/frobt.2021.758177)
Supplement: Supplementary file 3 [file DataSheet1.docx]

Supplementary Material

## Supplementary Appendices

# Appendix Ⅰ:

*Questionnaire to assess the sense of being attended (SoBA) to of the interviewee.*

| Item | Questions |
| --- | --- |
|  | The interviewee (Mr. A) felt that the interviewer (Mr. B): |
| 1 | carefully listened to his answer? |
| 2 | was interested in his answer? |
| 3 | was attentive to his answer? |
| 4 | was trying to understand his answer. |
| * Internal consistency α = 0.81. | |

# Appendix Ⅱ:

## Human condition (Human in Experiment-Ⅰ and Human (averted) in Experiment-Ⅱ):

In human conditions (see Fig. 02 and 07), the participants were discussing earning money through unfair means and paying taxes. “*Q*” represents the interviewer’s question and “*A*” represents the interviewee’s answer in his/her voice.

***Interviewer*:** *Q1*: If you get a chance to earn a money that is not 100 % fair near you, will you go for that? ***Interviewee*:** *A1*: No, I will not go for that. ***Interviewer*:** *Q2*: Why? Why will you not go for that? ***Interviewee*:** *A2*: Well, I think it is ethically not correct and it is important for me. So, this is the reason I will not go for that.

## Robot condition:

In the Robot condition of Experiment-Ⅰ (Fig. 03(b)) and Ⅱ (Fig. 08(b)), the participants were discussing the same issue as in the Human conditions. “Q” represents the interviewer’s question and “A” represents the interviewee’s answer, where the first answer is given by the teleoperated robot avatar, whereas the second answer is the interviewer’s own voice.

***Interviewer***: *Q1*: If you get a chance to earn a money that is not 100 % fair near you, will you go for that? ***Interviewee***: *A1*: No, I think I do not. (*Robot avatar utterance*). ***Interviewer***: *Q2*: Why? Why will you not go to this? You (pointing gesture toward the online conference monitor with web camera so that interviewee perceived that the pointing gesture was toward him/herself) can think with him (pointing gesture to robot avatar) and propose some answer. ***Interviewee*** *A2*: Well, I think it is ethically incorrect and it is important for me. This is the reason I will not go for that.
